# Predictive risk scores for visual prognosis after photodynamic therapy for central serous chorioretinopathy

**DOI:** 10.1007/s00417-024-06698-1

**Published:** 2024-11-22

**Authors:** Ryoh Funatsu, Hiroto Terasaki, Naohisa Mihara, Hideki Shiihara, Shozo Sonoda, Taiji Sakamoto

**Affiliations:** Department of Ophthalmology, Kagoshima University Graduate School of Medical and Dental Sciences, Kagoshima, Japan

**Keywords:** Central serous chorioretinopathy, Pachychoroid-spectrum disorders, Predictive factors, Optical coherence tomography

## Abstract

**Purpose:**

To comprehensively evaluate baseline characteristics of patients with central serous chorioretinopathy (CSC) and develop predictive risk scores to identify visual prognosis.

**Methods:**

This single-institute, retrospective cohort study included 144 eyes of 144 patients with CSC who underwent photodynamic therapy and achieved serous retinal detachment resolution. We developed and assessed the performance of several risk scores for best-corrected visual acuity (BCVA) outcomes six months post-treatment: i) BCVA improvement (≤-1.0 logMAR), and ii) BCVA deterioration (≥+ 1.0 logMAR).

**Results:**

The BCVA improvement models used photoreceptor outer segment thickness, loss of photoreceptor outer segment, and neurosensory retinal thickness (NSRT), while the BCVA deterioration models included outer nuclear layer thickness and NSRT. The BCVA improvement models demonstrated a corrected area under the curve (AUC) of 0.786 (95% confidence interval [CI]: 0.699–0.864), with 80.4% sensitivity, and 71.2% specificity. The BCVA deterioration models achieved a corrected AUC of 0.864 (95% CI: 0.742–0.958), with 85.7% sensitivity, and 83.5% specificity.

**Conclusion:**

The predictive models for CSC exhibited favorable performance in predicting individual visual prognoses. A thinner outer nuclear layer may be associated with BCVA deterioration, whereas preservation of the photoreceptor outer segment may be correlated with BCVA improvement.

**Key Messages:**

***What is known*:**

- Pre-treatment best-corrected visual acuity, thickness of each sensory retinal layer, time from onset to treatment, and macular atrophy were each found to be associated with visual prognosis for patients with central serous chorioretinopathy (CSC).

***What is new*:**

- The current study comprehensively assessed potential prognostic factors and precisely identified individual likelihood of visual prognosis.
- The study found that different regions of the sensory retina were associated with either worsening or improving visual acuity.
- Accurately predicting visual outcomes after photodynamic therapy for CSC would help healthcare providers create personalized treatment plans and enable patients to make informed decisions about their treatment based on their expected visual results.

**Supplementary Information:**

The online version contains supplementary material available at 10.1007/s00417-024-06698-1.

## Introduction

Central serous chorioretinopathy (CSC) is a disease characterized by serous retinal detachment (SRD) in the macula and predominantly affects working-age men [1, 2]. Some patients with chronic CSC present with persistent SRD and macular complications. Approximately 13% of patients with chronic CSC develop legal blindness in the long term, emphasizing the critical need for appropriate intervention to prevent vision loss in patients and society [3, 4]. 

Photodynamic therapy (PDT) is considered the most effective treatment for CSC [5–9], with existing studies indicating the correlation between earlier PDT intervention and superior visual outcomes [10–13]. However, given the gradual progression of vision loss in CSC [3], and the necessity for PDT, which mandates access to specialized equipment in secondary or tertiary medical facilities, coupled with posttreatment lifestyle restrictions [14, 15], a notable cohort of working-age patients may decline aggressive treatment and opt for observational management. This phenomenon poses a substantive impediment to achieving favorable visual outcomes in patients with CSC. Therefore, the accurate prediction of visual prognosis following PDT for CSC would facilitate healthcare providers in devising tailored treatment strategies, whereas empowering patients to make informed treatment choices regarding their course of treatment based on their anticipated visual outcomes.

Previous studies have reported several clinical findings associated with posttreatment visual acuity in CSC [10–12, 16–19]. However, comprehensive analyses of these factors to estimate the probability of visual prognosis for individual CSC patients are lacking. Especially, the potential predictive factors for visual acuity deterioration or improvement after PDT are not well understood.

This study aimed to develop a risk prediction model for visual prognosis in CSC patients by comprehensively analyzing potential predictive factors.

## Materials and methods

### Study design

This retrospective study included consecutive patients with CSC treated with PDT and followed up for at least 6 months (December 2015 to June 2023) at the Department of Ophthalmology, Kagoshima University Hospital. This study was approved by the Ethics Committee of Kagoshima University (no. 170283). All procedures were conducted according to the tenets of the Declaration of Helsinki.

## Participants

The diagnosis of CSC was made based on the criteria established in previous studies and was defined as an eye with SRD in the macula on optical coherence tomography (OCT) and leakage within the SRD on fluorescein angiography [5, 6, 10, 11]. Several patients included in this study were also reported in our previous publications [10, 11]. In cases where patients were unsuitable for fluorescein angiography, a retina specialist (HT) determined the diagnosis based on alternative findings. This study included patients with type 1 macular neovascularization as CSC with macular neovascularization. We excluded patients (i) with a history of PDT; (ii) with comorbidities, such as secondary macular edema unrelated to CSC or other causes of visual loss; (iii) who received additional treatment for CSC or underwent cataract surgery during follow-up; (iv) with blurred images; and (v) whose eyes experienced recurrent SRD after PD. If both eyes were included, the more recently treated eyes were selected. Two examiners (RF and NM) discussed and evaluated image quality based on two criteria: whether the center of foveal depression was visible in OCT B-scan images and the clarity of retinal layer boundaries. Images identified as blurred by the examiners were excluded from the analysis.

## Image evaluations

The following examinations were performed at the initial visit, unless the patient was allergic to contrast media: general medical interview (daily habits and medical history including treatment history), best-corrected visual acuity (BCVA) test (decimal visual acuity), anterior and posterior segment examination using slit-lamp biomicroscopy, refraction test (RM8900, Topcon, Tokyo, Japan), axial length measurement (OA-2000 Optical Biometer, Tomey, Tokyo, Japan), color fundus photography (DRI OCT Triton, Topcon, Tokyo, Japan), spectral-domain OCT (SPECTRALIS, Heidelberg Engineering, Heidelberg, Germany), fundus autofluorescence (FAF) (SPECTRALIS); and optical coherence tomography angiography (OCTA) (PLEX Elite 9000, ZEISS, Oberkochen, Germany); fluorescein angiography and indocyanine green angiography (SPECTRALIS). Information pertaining to CSC-related findings was gathered based on the reports of previous studies (Table S1) [3, 10, 11, 13, 18, 20–23]. The reliability of OCT thickness measurements has been previously documented [10, 11, 18]. Three authors (RF, NM, and HS) evaluated the qualitative items, each of whom had published at least three peer-reviewed articles on CSC. Disagreements among all three authors were resolved by consulting a supervisor (HT) or by majority vote. The FAF image of each eye was rated from 0 to 4 in the order of severity [23], with the means rounded to the nearest whole number. Inter-observer agreement was assessed using the adjusted kappa coefficient (AC1 statistic) due to the highly skewed prevalence of findings in certain items [24–26]. 

## Predicting model development and evaluation

This study evaluated the following models: (1) a model predicting visual acuity deterioration ( ≥ + 0.10 logMAR change from the baseline); and (2) a model predicting visual acuity improvement in eyes with pretreatment BCVA more than 0.00 logMAR ( ≤ − 0.10 logMAR change from the baseline). The predictive models were constructed using the following procedures: (i) Variable screening was performed by extracting items that were significantly associated with the categories (*P* < 0.05). (ii) The area under the curve (AUC) of the receiver operating characteristic curve was calculated, and the cutoff between categories (the point closest to the upper left) was determined for the selected continuous variables. If the lower limit of the 95% confidence interval (CI) of the AUC was > 0.5, the cutoff was used (Table S2). (iii) Using the selected variables, a forward-backward stepwise method was employed to determine the variables for regression analyses. Lastly, (iv) the score was calculated based on the estimated regression coefficients.

The AUC, sensitivity, specificity, positive predictive value, and negative predictive value of each prediction model were used to evaluate the model performance. The AUC was calculated using the bootstrap method with 1,000 bootstrap samples to correct for optimism, and Harrell’s method was employed to calculate the 95% CI.

### Statistical analysis

Given the small proportion of missing values in these data, a complete case analysis was performed after variable screening (smoking history, 12.5%; duration from the first episode, 2.8%; axial length, 2.1%; type of FAF, 2.1%; choroidal hyperpermeability, 0.1%; leakage, 0.1%; and others, 0.0%). Kruskal-Wallis rank sum test, Mann-Whitney U test for continuous variables, and Fisher’s exact test for categorical variables were used to compare the pretreatment factors across categories. Logistic regression analysis was used to examine the prediction models for the visual prognosis. Given the separation issue, Firth’s bias correction was applied in the logistic regression analysis [27]. Prediction scores were calculated by rounding the estimated regression coefficients of the selected variables based on a previous report [28]. The significance level was set at a P value of 0.05, and the R software (version 4.3.2) was used to perform all analyses.

## Results

### Patient characteristics

A total of 144 eyes of 144 patients with CSC were examined. Table 1 and Table S3 showed the detailed patient characteristics. The mean age was 58.9 ± 11.04 years, and 81.9% of patients were male. In addition, mean duration from the first episode was 42.33 ± 68.37 months. A total of 14 eyes (9.7%) comprised the BCVA deteriorated group. Of the 106 eyes with BCVA > 0.0 logMAR, 52 (49.1%) were classified as BCVA-improved.

**Table 1:** Patient characteristics of study participants

Characteristics	*N* = 144
Male, n (%)	118 (81.9)
Mean age (SD); median, year	58.92 ± 11.04; 58.00
Duration from the first episode (SD); median, month	42.33 ± 68.37; 14.50
Treatment history, n (%)	
Anti-VEGF drugs	35 (24.3)
Photocoagulation	14 (9.7)
None	95 (66.0)
Mean BCVA (SD); median, logMAR	0.20 ± 0.25; 0.15
Mean SFRT (SD); median, µm	298.94 ± 95.61; 286.00
Mean NSRT (SD); median, µm	161.35 ± 48.80; 162.00
Mean ONLT (SD); median, µm	85.84 ± 24.04; 86.00
Mean ELM**-**bottom of photoreceptor thickness (SD); median, µm	75.51 ± 35.78; 68.00
Mean SFCT (SD); median, µm	402.43 ± 133.03; 385.50
Elongation of photoreceptor outer segment, n (%)	118 (81.9)
Loss of photoreceptor outer segment, n (%)	24 (16.7)
Disorganization of external limiting membrane, n (%)	51 (35.4)
Macular neovascularization, n (%)	38 (26.4)
Classification of fundus autofluorescence (≤ Hypo), n (%)	43 (30.5)

*SD *standard deviation, *VEGF* vascular endothelial growth factor, *BCVA* best-corrected visual acuity, *SFRT* subfoveal retinal thickness, *NSRT* neurosensory retinal thickness, *ONLT* outer nuclear layer thickness, *ELM* external limiting membrane, *SFCT* subfoveal choroidal thickness

The AC1 statistics for reduced fundus tessellation, FAF classifications, external limiting membrane (ELM) disorganization, microrips of retinal pigment epithelium, and hyperreflective foci showed moderate reproducibility, and the other features demonstrated good agreement (Table S4).

## Development of predictive risk scores

Significant differences were observed between the BCVA-improved group and the BCVA-not-improved group in six parameters: elongation of photoreceptor outer segment (improved vs. not-improved, 90.4% vs. 68.5%, *P* = 0.008), loss of photoreceptor outer segment (improved vs. not-improved, 5.8% vs. 33.3%, *P* < 0.001), FAF classification: Hypo or not (improved vs. not-improved, 21.6% vs. 44.2%, *P* = 0.021), outer nuclear layer thickness (ONLT) (improved vs. not-improved, 89.27 ± 36.26 μm vs. 75.04 ± 29.11 μm, *P* < 0.001), external limiting membrane (ELM)-bottom of photoreceptor thickness (improved vs. not-improved, 83.27 ± 36.63 μm vs. 56.57 ± 24.18 μm, *P* < 0.001), and neurosensory retinal thickness (NSRT) (improved vs. not-improved, 172.54 ± 40.17 μm vs. 131.61 ± 46.43 μm, *P* < 0.001, Table S5).

Significant differences were observed between the BCVA-deteriorated group and BCVA-non-deteriorated group in seven parameters: loss of photoreceptor outer segment (deteriorated vs. not-deteriorated, 42.9% vs. 13.9%, *P* = 0.014), FAF classification: Hypo or not (deteriorated vs. not-deteriorated, 64.3% vs. 26.8%, *P* = 0.011), BCVA (deteriorated vs. not-deteriorated, 0.41 ± 0.27 logMAR vs. 0.17 ± 0.24 logMAR, *P* = 0.001), ONLT (deteriorated vs. not-deteriorated, 62.79 ± 18.56 μm vs. 88.32 ± 23.29 μm, *P* < 0.001), ELM-bottom of photoreceptor thickness (deteriorated vs. not-deteriorated, 43.29 ± 15.11 μm vs. 78.98 ± 35.65 μm, *P* < 0.001), and NSRT (deteriorated vs. not-deteriorated, 106.07 ± 25.23 μm vs. 167.31 ± 47.00 μm, *P* < 0.001, Table S5).

ELM-bottom of photoreceptor thickness (β = 0.85), loss of photoreceptor outer segmen (β = −0.93), and NSRT (β = 1.20) were selected as variables for risk score components of BCVA improvement, while ONLT (β = 1.14) and NSRT (β = 2.44) were selected for predicting BCVA deterioration (Table 2).

**Table 2:** Selected variables and scores for predicting BCVA change after photodynamic therapy for central serous choriortetinopathy

BCVA improvement			BCVA deterioration		
	Regression coefficients	Risk Scores		Regression coefficients	Risk Scores
NSRT < 153.5 μm ≥ 153.5 μm	Ref. 1.20	0 1	NSRT ≥ 125.5 μm < 125.5 μm	Ref. 2.44	0 2
ELM-bottom of photoreceptor thickness < 68.5 μm ≥ 68.5 μm	Ref. 0.85	0 1	ONLT ≥ 62.0 μm < 62.0 μm	Ref. 1.14	0 1
Loss of photoreceptor outer segment	−0.93	−1			

Regression coefficients were calculated using Firth’s bias correction. *BCVA* best-corrected visual acuity, *NSRT* neurosensory retinal thickness, *ONLT* outer nuclear layer thickness, *ELM* external limiting membrane

### Model performance

**Fig. 1:**
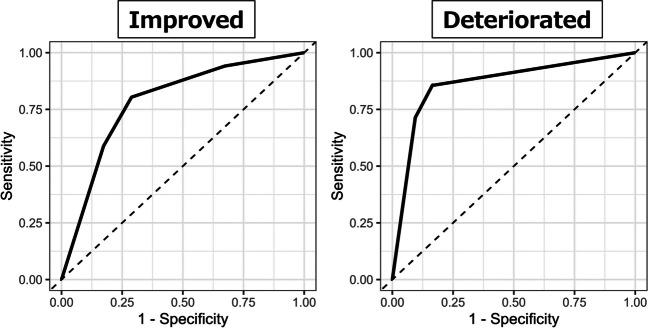
The ROC curves of each prediction model for visual prognosis: visual improvement and deterioration The model for predicting BCVA improvement demonstrated the corrected AUC was 0.786, while the model for BCVA deterioration showed the corrected AUC was 0.864

To predict BCVA improvement, the corrected AUC of the predictive score was 0.786 (95% CI: 0.699–0.864), with a sensitivity of 80.4% and a specificity of 71.2% (Fig. 1; Table 3). In the cohort with the highest risk score for predicting BCVA improvement, 76.9% of patients demonstrated actual BCVA improvement.

**Table 3:** Performance of models for predicting BCVA improvement or deterioration after photodynamic therapy

BCVA improvement		BCVA deterioration	
**Score**		**Score**	
**−1**	3/20 (15.0%)	**0**	2/107 (1.9%)
**0**	7/27 (25.9%)	**1**	0/1 (0.0%)
**1**	11/17 (64.7%)	**2**	2/11 (18.2%)
**2**	30/39 (76.9%)	**3**	10/22 (45.5%)
AUC*	0.786 (95%CI 0.699–0.864)	AUC*	0.864 (95%CI 0.742–0.958)
**Cut-off**	≥ 1	**Cut-off**	≥ 2
**Sensitivity**	80.4%	**Sensitivity**	85.7%
**Specificity**	71.2%	**Specificity**	83.5%
**PPV**	73.2%	**PPV**	36.4%
**NPV**	78.7%	**NPV**	98.1%

*AUC is corrected using bootstrapping analysis. *BCVA* best-corrected visual acuity, *AUC* area under the curve, *CI* confidence interval, *PPV* positive predictive value, *NPV* negative predictive values

When predicting BCVA deterioration, the model achieved a corrected AUC of 0.864 (95% CI: 0.742–0.958), with a sensitivity of 85.7% and a specificity of 83.5% (Table 3). In this cohort with the highest risk score for predicting BCVA deterioration, 45.5% of patients demonstrated actual BCVA deterioration.

## Discussion

The current study developed a clinical prediction model to accurately estimate the risk of different visual outcomes following PDT for CSC. The study also identified potential prognostic factors for improved visual acuity and worsening visual acuity. Importantly, it revealed that the layer of the neurosensory retina that are important for prediction vary between improving and worsening visual acuity. Moreover, the identified factors are easily obtainable, making these models practical for daily use.

The preservation of the photoreceptor layer is identified as a potential factor associated with visual acuity improvement following PDT for CSC [17]. In contrast, the factors associated with visual acuity loss, such as NSRT and ONLT, have been reported in previous studies [11]. Nevertheless, the extent to which these factors can accurately predict vision changes in patients with CSC remains unclear. In our study, we comprehensively investigated the pretreatment factors for visual acuity changes and identified photoreceptor layer thickness as a potential prognostic factor for visual acuity improvement, in addition to the previously recognized factors. Furthermore, our findings suggest that distinct layers of the neurosensory retina may play differential roles in predicting BCVA improvement versus deterioration.　Using these neurosensory retinal layer’s findings, a high accuracy was achieved in predicting each visual prognosis.

Animal models have shown that retinal detachment induces photoreceptor cell ischemia, which initially leads to photoreceptor degeneration, followed by a gradual decrease and ultimate disappearance of photoreceptors [29–31]. Although the extent to which OCT detects this histologic change remains unclear, ONL thinning may signify photoreceptor cell decline, while photoreceptor outer segment loss or thinning may indicate a progression from photoreceptor cell degeneration to loss. Moreover, histological studies have demonstrated that PDT causes changes in the retina and choroid, including choriocapillaris occlusion and ONL thinning [32, 33]. Thus, eyes with ONL thinning may have a limited reserve of surviving photoreceptor cells and a reduced tolerance to PDT-induced photoreceptor cell damage, making OCT-measured ONL thickness a potential predictor of visual acuity loss following PDT. Furthermore, eyes with photoreceptor outer segment thinning or loss may have substantially fewer or no viable photoreceptor cells, potentially indicating these findings could have predicted visual acuity improvement. In other words, ONL morphology may reflect the resilience of the neurosensory retina, while photoreceptor outer segment morphology may indicate the retina’s potential for recovery.

Consistent with previous reports, our result showed that PDT is effective in CSC patients regardless of MNV presence [34, 35]. Furthermore, despite the relatively low complication rate associated with PDT for CSC [6, 7, 10, 11, 36], recommending prophylactic treatment to patients with relatively good visual acuity poses more significant challenges than intervening after the vision begins to deteriorate. The potential for spontaneous resolution of SRD in patients with chronic CSC without treatment may also prolong the follow-up period, even after vision loss occurs [9]. Therefore, predicting the probability of visual prognosis for each individual will provide additional information to support decision-making regarding therapeutic interventions. We developed prediction models using regression analysis, incorporating data exclusively from the pretreatment phase. These data were obtained through widely used medical examinations and patient interviews. The models offer interpretability by examining the risk scores of each factor. Considering these aspects, the models developed in this study may be practical for clinical applications. Thus, our models have the potential to facilitate appropriate therapeutic interventions.

This study has several limitations. First, it was a retrospective observational study with a relatively small sample size. Another limitation was the use of qualitative variables. However, we examined the reproducibility and analyzed only the quantitative indicators, ensuring that the findings remained largely consistent. Although the scores in this study underwent internal validation using the bootstrap method, their generalizability to other populations remains unclear and warrants further investigation in future studies. Without data on the number of exudative episodes and actual duration of serous detachment, which might be potential predictors of visual prognosis, the effect of time since the first episode may be underestimated. We emphasize the following two points: first, that the main objective of this study was to develop a predictive model, and second, that exclusion from the model does not rule out a variable’s potential causal relationship.

In conclusion, the models developed in this study effectively predicted visual acuity and the changes in visual acuity after PDT in patients with CSC while maintaining feasibility and interpretability. Utilizing these models to determine an individual’s likelihood of visual prognosis can facilitate personalized treatment for each patient with CSC.

## Electronic supplementary material

Below is the link to the electronic supplementary material.

Supplementary Material 1

Supplementary Material 2

Supplementary Material 3

Supplementary Material 4

Supplementary Material 5
