# Supplementary material for: Predictive risk scores for visual prognosis after photodynamic therapy for central serous chorioretinopathy
Source: Graefes Arch Clin Exp Ophthalmol. 2024 Nov 22;263(3):705–11. doi: 10.1007/s00417-024-06698-1 (PMC11953169; doi:10.1007/s00417-024-06698-1)
Supplement: Supplementary file 1 — Supplementary Material 1 [file 417_2024_6698_MOESM1_ESM.docx]

Table S4. Inter-rater agreement on subjective assessments among the three examiners.

| **Characteristics** | **Agreement percentage** | **AC1 statistics (Adjusted Kappa coefficients)** | **95% confidence interval** |
| --- | --- | --- | --- |
| Reduced fundus tessellation | 63.2% | 0.543 | 0.434–0.653 |
| Elongation of POS | 67.4% | 0.669 | 0.572–0.765 |
| Loss of POS | 81.2% | 0.817 | 0.746–0.888 |
| Disorganization of ELM | 59.7% | 0.518 | 0.401–0.635 |
| CMD | 96.5% | 0.976 | 0.955–0.998 |
| Microrip | 63.2% | 0.557 | 0.443–0.671 |
| HRF | 63.2% | 0.589 | 0.481–0.696 |
| Pachyvessel | 86.8% | 0.896 | 0.847–0.945 |
| FAF class | 44.4% | 0.529 | 0.457–0.600 |
| Macular atrophy | 79.7% | 0.816 | 0.745–0.886 |
| CVH | 76.2% | 0.841 | 0.730–0.869 |
| FA leakage: Diffuse vs Focal | 55.6% | 0.610 | 0.537–0.683 |
| Asymmetry type | 66.0% | 0.660 | 0.581–0.738 |
| Anastomosis | 84.0% | 0.864 | 0.805–0.923 |
| Pachychoroid or not | 65.3% | 0.714 | 0.641–0.788 |

**Abbreviation:** **POS**, photoreceptor outer segment; **ELM**, external limiting membrane; **CMD**, cystoid macular degeneration; **HRF**, hyperreflective foci; **FAF**, fundus autofluorescence; **CVH**, choroidal vascular hyperpermeability **FA,** fluorescein angiography.
