# Supplementary material for: Predictive risk scores for visual prognosis after photodynamic therapy for central serous chorioretinopathy
Source: Graefes Arch Clin Exp Ophthalmol. 2024 Nov 22;263(3):705–11. doi: 10.1007/s00417-024-06698-1 (PMC11953169; doi:10.1007/s00417-024-06698-1)
Supplement: Supplementary file 2 — Supplementary Material 2 [file 417_2024_6698_MOESM2_ESM.docx]

Table S1. The qualitative findings related to central serous chorioretinopathy collected in this study

| **Qualitative findings** | **Examinations** | **Categories** |
| --- | --- | --- |
| The reduced fundus tessellation | Color fundus photography | Present or not |
| The elongation of photoreceptor outer segment | Optical coherence tomography (One slice, 30°, center of the fovea, horizontal, image averaging was set at 100) | Present or not |
| The loss of photoreceptor outer segment | Optical coherence tomography (One slice, 30°, center of the fovea, horizontal, image averaging was set at 100) | Present or not |
| The disorganization of external limiting membrane | Optical coherence tomography (One slice, 30°, center of the fovea, horizontal, image averaging was set at 100) | Present or not |
| The micro rip | Optical coherence tomography (volume scan, 30 × 25°, center of the fovea, horizontal, image averaging was set at 100) | Present or not |
| The hyperreflective foci | Optical coherence tomography (volume scan, 30 × 25°, center of the fovea, horizontal, image averaging was set at 100) | Present or not |
| The cystoid macular degeneration | Optical coherence tomography (volume scan, 30 × 25°, center of the fovea, horizontal, image averaging was set at 100) | Presence involving the fovea; Presence without involving the fovea; Not present |
| The Haller’s vessel running patterns | Optical coherence tomography (12 × 12mm, en-face) and/or indocyanine angiography | The symmetry type; the upper dominant type; the lower dominant type |
| The anastomosis of Haller’s vessel | Optical coherence tomography (12 × 12mm, en-face) and/or indocyanine angiography | Present or not |
| Pachyvessel | Optical coherence tomography (12 × 12mm, en-face, B scan [enhanced depth imaging]) and/or indocyanine angiography | Present or not |
| Macular neovascularization | Optical coherence tomography angiography (6 × 6mm) and/or fluorescein angiography and/or indocyanine angiography | Present or not |
| Macular atrophy | Optical coherence tomography and/or color fundus photography and/or fundus autofluorescence | Present or not |
| Classification of Fundus autofluorescence | Fundus autofluorescence | Non-hypo class: blocked; mottled; hyper, and the hypo class: the hyper/hypo; descending tract |
| Choroidal hyperpermeability | Indocyanine angiography | Present or not |
| Leakage | Fluorescein angiography | Diffuse or focal |
| Pachychoroid or not | Comprehensive decision | With confidence; with suspicion; does not appear to be pachychoroid |
| Smoking history | Medical questionnaire | Never (0 pack-year); Moderate (1–19 pack years); Heavy (≥ 20 pack-years) |
