# Supplementary material for: Predictive risk scores for visual prognosis after photodynamic therapy for central serous chorioretinopathy
Source: Graefes Arch Clin Exp Ophthalmol. 2024 Nov 22;263(3):705–11. doi: 10.1007/s00417-024-06698-1 (PMC11953169; doi:10.1007/s00417-024-06698-1)
Supplement: Supplementary file 3 — Supplementary Material 3 [file 417_2024_6698_MOESM3_ESM.docx]

Table S2. Categorization of the screened continuous variables using calculated cut-off values

| **Characteristics** | **Improved or not** | **Deteriorated or not** |
| --- | --- | --- |
| BCVA | - | 0.26 logMAR  AUC: 0.627–0.900 |
| NSRT | 153.5 μm  AUC: 0.668–0.853 | 125.5 μm  AUC: 0.815–0.947 |
| ONLT | 78.5 μm  AUC: 0.589–0.795 | 62.0 μm  AUC: 0.686–0.934 |
| ELM- bottom of photoreceptor thickness | 68.5 μm  AUC: 0.642–0.832 | 53.5 μm  AUC: 0.756–0.935 |

AUC was shown in a 95 % confidence interval. **Abbreviation: BCVA**, best-corrected visual acuity; **SFRT**, subfoveal retinal thickness; **NSRT**, neurosensory retinal thickness; **ONLT**, outer nuclear layer thickness; **ELM**, external limiting membrane.
