# Supplementary material for: Predictive risk scores for visual prognosis after photodynamic therapy for central serous chorioretinopathy
Source: Graefes Arch Clin Exp Ophthalmol. 2024 Nov 22;263(3):705–11. doi: 10.1007/s00417-024-06698-1 (PMC11953169; doi:10.1007/s00417-024-06698-1)
Supplement: Supplementary file 4 — Supplementary Material 4 [file 417_2024_6698_MOESM4_ESM.docx]

Table S3. Patient characteristics of study population

| **Characteristics** | N = 144 |  |
| --- | --- | --- |
| Mean axial length (SD); median, mm | 23.55 ± 1.06; 23.53 |  |
| Smoking history, n (%) |  |  |
| Heavy | 40 (31.8) |  |
| Moderate | 15 (11.9) |  |
| Never | 71 (56.4) |  |
| Unknown | 18 |  |
| Mean SRF height (SD); median, μm | 137.59 ± 82.26; 119.50 |  |
| Choroidal vessel anastomosis, n (%) | 127 (88.2) |  |
| Running patterns of Haller’s vessel, n (%) |  |  |
| Upper dominant | 49 (34.0) |  |
| Lower dominant | 48 (33.3) |  |
| Symmetry | 47 (32.6) |  |
| Pachyvessel, n (%) | 135 (93.8) |  |
| Choroidal hyperpermeability, n (%) | | 134 (93.7) |
| Unknown | 1 |  |
| Fluorescein angiography: leakage patterns, n (%) |  |  |
| Diffuse | 97 (67.8) |  |
| Focal | 46 (32.2) |  |
| Unknown | 1 |  |
| Reduced fundus tessellation, n (%) | 94 (65.3) |  |
| Cystoid macular degeneration, n (%) | 2 (1.4) |  |
| Microrip of retinal pigment epithelium, n (%) | 54 (37.5) |  |
| Hyperreflective foci, n (%) | 108 (75.0) |  |
| Classification of fundus autofluorescence, n (%) |  |  |
| Blocked | 19 (13.5) |  |
| Mottled | 46 (32.6) |  |
| Hyper | 33 (23.4) |  |
| Hyper/Hypo | 24 (17.0) |  |
| Descending tract | 19 (13.5) |  |
| Unknown | 3 |  |
| Macular atrophy, n (%) | 16 (11.1) |  |
| Pachychoroid or not, n (%) |  |  |
| With confidence | 116 (80.6) |  |
| With suspicion | 23 (16.0) |  |
| Does not appear to | 5 (3.5) |  |

**Abbreviation: SD**, standard deviation**; SRF**, subretinal fluid.
