# Supplementary material for: Predictive risk scores for visual prognosis after photodynamic therapy for central serous chorioretinopathy
Source: Graefes Arch Clin Exp Ophthalmol. 2024 Nov 22;263(3):705–11. doi: 10.1007/s00417-024-06698-1 (PMC11953169; doi:10.1007/s00417-024-06698-1)
Supplement: Supplementary file 5 — Supplementary Material 5 [file 417_2024_6698_MOESM5_ESM.docx]

Table S5. Comparisons of pre-treatment characteristics stratified by improvement or deterioration in BCVA after photodynamic therapy

| **Characteristics** | **BCVA-not-improved**  N = 54 | **BCVA-improved**  N = 52 | **P value** | **BCVA-not-deteriorated**  N = 130 | **BCVA-deteriorated**  N = 14 | **P value** |
| --- | --- | --- | --- | --- | --- | --- |
| Male, n (%) | 46 (85.2) | 40 (76.9) | 0.326 | 108 (83.1) | 10 (71.4) | 0.282 |
| Mean age (SD); median, year | 61.87 ± 11.20; 61.50 | 60.06 ± 10.55; 61.00 | 0.600 | 59.20 ± 11.32; 59.00 | 56.36 ± 7.86; 54.00 | 0.277 |
| Duration from the first episode (SD); median, month | 51.63 ± 75.10; 17.00 | 37.21 ± 73.41; 13.00 | 0.102 | 40.75 ± 68.85; 14.00 | 57.77 ± 63.97; 36.00 | 0.134 |
| Treatment history |  |  | 0.672 |  |  | 1.000 |
| Anti-VEGF drugs | 14 (25.9%) | 14 (26.9%) |  | 32 (24.6%) | 3 (21.4%) |  |
| Photocoagulation | 3 (5.6%) | 5 (9.6%) |  | 85 (65.4%) | 10 (71.4%) |  |
| None | 37 (68.5%) | 33 (63.5%) |  | 13 (10.0%) | 1 (7.1%) |  |
| Mean BCVA (SD); median, logMAR | 0.33 ± 0.27; 0.22 | 0.23 ± 0.19; 0.15 | 0.097 | 0.17 ± 0.24; 0.13 | 0.41 ± 0.27; 0.46 | 0.001 |
| Mean SFRT (SD); median, μm | 276.94 ± 101.49; 259.50 | 298.46 ± 83.70; 284.50 | 0.180 | 302.97 ± 96.88; 281.50 | 261.57 ± 75.93; 295.50 | 0.154 |
| Mean NSRT (SD); median, μm | 131.61 ± 46.43; 122.00 | 172.54 ± 40.17; 166.50 | < 0.001 | 167.31 ± 47.00; 166.50 | 106.07 ± 25.23; 105.50 | < 0.001 |
| Mean ONLT (SD); median, μm | 75.04 ± 29.11; 70.50 | 89.27 ± 17.26; 88.00 | < 0.001 | 88.32 ± 23.29; 88.50 | 62.79 ± 18.56; 57.50 | < 0.001 |
| Mean ELM**-**bottom of photoreceptor thickness (SD); median, μm | 56.57 ± 24.18; 52.00 | 83.27 ± 36.63; 78.00 | < 0.001 | 78.98 ± 35.65; 72.50 | 43.29 ± 15.11; 43.50 | < 0.001 |
| Mean SFCT (SD); median, μm | 398.24 ± 148.29; 376.00 | 389.13 ± 130.43; 377.50 | 0.786 | 404.08 ± 128.94; 387.50 | 387.07 ± 171.50; 370.00 | 0.383 |
| Elongation of photoreceptor outer segment, n (%) | 37 (68.5) | 47 (90.4) | 0.008 | 109 (83.9) | 9 (64.3) | 0.134 |
| Loss of photoreceptor outer segment, n (%) | 18 (33.3) | 3 (5.8) | < 0.001 | 18 (13.9) | 6 (42.9) | 0.014 |
| Disorganization of external limiting membrane, n (%) | 24 (44.4) | 20 (38.5) | 0.560 | 44 (33.9) | 7 (50.0) | 0.250 |
| Macular neovascularization, n (%) | 37 (68.5) | 37 (71.2) | 0.834 | 34 (26.2) | 4 (28.6) | 1.000 |
| Mean axial length (SD); median, mm | 23.57 ± 0.98; 23.74 | 23.64 ± 1.23; 23.53 | 0.937 | 23.53 ± 1.05; 23.51 | 23.71 ± 1.16; 23.80 | 0.510 |
| Smoking history, n (%) |  |  | 0.254 |  |  | 0.919 |
| Heavy | 14 (30.4) | 19 (41.3) |  | 37 (32.5) | 3 (25.0) |  |
| Moderate | 3 (6.5) | 6 (13.0) |  | 14 (12.3) | 1 (8.3) |  |
| Never | 29 (63.0) | 21 (45.7) |  | 63 (55.3) | 8 (66.7) |  |
| Unknown | 8 | 6 |  | 16 | 2 |  |
| Mean SRF height (SD); median, μm | 145.33 ± 88.40; 119.50 | 125.92 ± 71.59; 113.50 | 0.337 | 135.66 ± 83.52; 117.50 | 155.50 ± 69.54; 168.00 | 0.212 |
| Choroidal vessel anastomosis, n (%) | 50 (92.6) | 42 (80.8) | 0.090 | 113 (86.9) | 14 (100) | 0.375 |
| Running patterns of Haller’s vessel, n (%) |  |  | 0.896 |  |  | 1.000 |
| Upper dominant | 20 (37.0) | 20 (38.5) |  | 44 (33.9) | 5 (35.7) |  |
| Lower dominant | 18 (33.3) | 15 (28.9) |  | 43 (33.1) | 5 (35.7) |  |
| Symmetry | 16 (29.6) | 17 (32.7) |  | 43 (33.1) | 4 (28.6) |  |
| Pachyvessel, n (%) | 52 (96.3) | 45 (86.5) | 0.090 | 122 (93.9) | 13 (92.9) | 1.000 |
| Choroidal hyperpermeability, n (%) | 49 (92.5) | 49 (94.2) | 1.000 | 121 (93.1) | 13 (100) | 1.000 |
| Unknown | 1 | 0 |  | 0 | 1 |  |
| Fluorescein angiography: leakage patterns, n (%) |  |  | 0.081 |  |  | 0.379 |
| Diffuse | 38 (70.4) | 38 (73.1) |  | 89 (69.0) | 8 (57.1) |  |
| Focal | 16 (29.6) | 14 (26.9) |  | 40 (31.0) | 6 (42.9) |  |
| Unknown | 0 | 0 |  | 1 | 0 |  |
| Reduced fundus tessellation, n (%) | 38 (70.4) | 31 (59.6) | 0.309 | 85 (65.4) | 9 (64.3) | 1.000 |
| Cystoid macular degeneration, n (%) | 1 (1.9) | 1 (1.9) | 1.000 | 2 (1.5) | 0 (0.0) | 1.000 |
| Microrip of retinal pigment epithelium, n (%) | 22 (40.7) | 18 (34.6) | 0.553 | 49 (37.7) | 5 (35.7) | 1.000 |
| Hyperreflective foci, n (%) | 39 (72.2) | 44 (84.6) | 0.159 | 96 (73.9) | 12 (85.7) | 0.518 |
| Classification of fundus autofluorescence, n (%) |  |  | 0.089 |  |  | 0.013 |
| Blocked | 5 (9.6) | 9 (17.7) |  | 19 (15.0) | 0 (0.0) |  |
| Mottled | 12 (23.1) | 21 (41.1) |  | 45 (35.4) | 1 (7.1) |  |
| Hyper | 12 (23.1) | 10 (19.6) |  | 29 (22.8) | 4 (28.6) |  |
| Hyper/Hypo | 12 (23.1) | 7 (13.7) |  | 20 (15.8) | 4 (28.6) |  |
| Descending tract | 11 (21.2) | 4 (7.8) |  | 14 (11.0) | 5 (35.7) |  |
| Unknown | 2 | 1 |  | 3 | 0 |  |
| Classification of fundus autofluorescence (≤ Hypo), n (%) | 23 (44.2) | 11 (21.6) | 0.021 | 34 (26.8) | 9 (64.3) | 0.011 |
| Unknown | 2 | 1 |  | 3 | 0 |  |
| Macular atrophy, n (%) | 10 (18.5) | 3 (5.8) | 0.073 | 13 (10.0) | 3 (21.4) | 0.190 |
| Pachychoroid or not, n (%) |  |  | 0.601 |  |  | 1.000 |
| With confidence | 44 (81.5) | 39 (75.0) |  | 104 (80.0) | 12 (85.7) |  |
| With suspicion | 9 (16.7) | 10 (19.2) |  | 21 (16.2) | 2 (14.3) |  |
| Does not appear to | 1 (1.9) | 3 (5.8) |  | 5 (3.9) | 0 (0.0) |  |

**Abbreviation: BCVA**, best-corrected visual acuity; **SD**, standard deviation**;** **VEGF,** vascular endothelial growth factor; **SFRT**, subfoveal retinal thickness; **NSRT**, neurosensory retinal thickness; **ONLT**, outer nuclear layer thickness; **ELM**, external limiting membrane; **SFCT**, subfoveal choroidal thickness; **SRF**, Subretinal fluid.
